# Update on Circulating Tumor Cells in Genitourinary Tumors with Focus on Prostate Cancer

**DOI:** 10.3390/cells9061495

**Published:** 2020-06-19

**Authors:** Alessia Cimadamore, Gaetano Aurilio, Franco Nolé, Francesco Massari, Marina Scarpelli, Matteo Santoni, Antonio Lopez-Beltran, Liang Cheng, Rodolfo Montironi

**Affiliations:** 1 Section of Pathological Anatomy, Faculty of Medicine, Polytechnic University of the Marche Region, United Hospitals, 60126 Ancona, Italy; m.scarpelli@univpm.it (M.S.); r.montironi@univpm.it (R.M.); 2 Department of Medical Oncology, Division of Urogenital and Head and Neck Tumours, IEO, European Institute of Oncology IRCCS, 20141 Milan, Italy; gaetano.aurilio@ieo.it (G.A.); franco.nole@ieo.it (F.N.); 3 Division of Oncology, S. Orsola-Malpighi Hospital, 40138 Bologna, Italy; fmassari79@gmail.com; 4 Oncology Unit, Macerata Hospital, 62100 Macerata, Italy; mattymo@alice.it; 5 Department of Surgery, Medical School, Cordoba University, 14004 Cordoba, Spain; em1lobea@gmail.com; 6 Department of Pathology and Laboratory Medicine, School of Medicine, Indiana University, Indianapolis, IN 462020, USA; liang_cheng@yahoo.com

**Keywords:** liquid biopsy, circulating tumor cells, genitourinary cancers, precision oncology, immune checkpoint inhibitors

## Abstract

Current developments in the treatment of genitourinary tumors underline the unmet clinical need for biomarkers to improve decision-making in a challenging clinical setting. The detection of circulating tumor cells (CTCs) has become one of the most exciting and important new approaches to identifying biomarkers at different stages of disease in a non-invasive way. Potential applications of CTCs include monitoring treatment efficacy and early detection of progression, selecting tailored therapies, as well as saving treatment costs. However, despite the promising implementation of CTCs in a clinical scenario, the isolation and characterization of these cells for molecular studies remain expensive with contemporary platforms, and significant technical challenges still need to be overcome. This updated, critical review focuses on the state of CTCs in patients with genitourinary tumor with focus on prostate cancer, discussing technical issues, main clinical results and hypothesizing potential future perspectives in clinical scenarios.

## 1. Introduction

Although promising improvements have been made in managing genitourinary cancers thanks to the discovery of emerging targets along with novel molecules, medical oncologists continue to suffer from the lack of valid tools for cancer diagnosis and treatment monitoring. There is an urgent need to finding biomarkers for prognostic and predictive use in genitourinary cancers patients. For this purpose, peripheral blood and urine would allow multiple serial sampling in a more convenient way at any stage of disease development, avoiding routine tissue-based samples usually associated with discomfort for the patients.

The term “liquid biopsy” traditionally identifies the use of blood-based analyses and other human fluids as surrogate materials of tissue/cytological samples. In other words, it can reasonably be considered as a process for identifying molecular circulating signatures shared with solid tumors. The research in liquid biopsy is currently focusing on circulating tumor cells (CTCs), which are rare nucleated cells originating from a malignancy or by metastases, circulating tumor DNA (ctDNA), the genetic material shed from primary tumor or metastases in the circulation, circulating cell-free RNA and microRNAs, extracellular vesicles and their content [1].

CTCs are a heterogeneous population (stem cells, progenitor cells, epithelial lineage cells) with regard to the proliferation rate and mutational burden and, as a consequence, are characterized by different aggressiveness. Through the peripheral blood, CTCs can extravasate and colonize distant sites to prime a metastatic process, and as such a self-perpetuating vicious cycle may be hypothesized [1,2].

Contrary to other liquid biopsy components, CTCs offer the possibility to perform assays like whole exome sequencing, splice variants analysis, RNA expression analysis, and functional assays. CTCs can also be cultured to assess drug resistance in vitro or in vivo and to create CTC-derived xenograft (CDX) models to mirror the tumor of the donor patient. Moreover, evaluation of signal colocalization is feasible only on CTCs. However, a small number of isolated CTCs is scarcely able to fully recapitulate the spatial and temporal tumor heterogeneity, a feature more attributable to ctDNA/RNA. Isolation of CTCs is technically harder compared to ctDNA and often limited by the extremely small number of CTCs in patient blood (1–100 cells per mL), varying among tumor types [1,2,3].

Over the years CTCs have been largely investigated postulating their introduction in clinical practice on the basis of different reasons: (i) ease of applicability; (ii) early diagnosis of inefficacy treatment subsequent to radical therapeutic approaches; (iii) classification of patients in prognostic groups based on post-therapy CTC persistence or decreasing; (iv) increasing knowledge into the metastatic development, as well as mechanisms of resistance and tumor response; (v) identification of genetic signature and characterization of the immune infiltrate and phenotypes that might be predictive of response to certain types of therapy.

Most of the literature data assessing the role of CTCs in a broad variety of tumor types have been mainly achieved using the CellSearch System, a semi-automated cytometric method, that has been shown to be reproducible with minimal intrapatient and interlaboratory variability, and to date is the only Food and Drug Administration (FDA)-approved test [2]. In 2008 the CellSearch System was granted FDA approval to aid in monitoring metastatic prostate cancer patients. So far, a great number of peer-reviewed articles have been published on CTCs and prostate cancer (PCa). The great interest in PCa is mostly due to the fact that its incidence is far more common compared to other genitourinary tumors. Clinically, there was a pressing need to find alternative methods to analyze metastatic tissues that, in the case of PCa, are commonly located in the bone, a difficult site to perform a biopsy.

Furthermore, under the umbrella of CTC category, detection of CTC clusters (also called circulating tumor microemboli (CTM) or mixed cells clusters) has aroused great interest in the research community. They were identified more than two decades ago in colon cancer and prostate cancer patients [3,4]. Their detection is based on the capture method where CTC surface antigens might underestimate the real CTM number in the blood stream. The aggregation of multiple cells, both cancer cells and non-cancer cells, such as immune cells and platelets, could impair and mask surface proteins’ exposure [5]. The clustering confers to CTCs shear stress resistance, apoptosis resistance, enhances their stemness, and favors immune escape [6]. Presence of CTMs might be predictive of cancer dissemination and resistance to treatments [7].

After the recent introduction of immune checkpoint inhibitors (ICIs) in several solid tumors, current investigations are exploring relevant immune-based biomarkers with CTCs in patients affected by genitourinary malignancies during treatment with ICIs (NCT02978118).

Based on these findings, we review the role of CTCs in prostate cancer (PCa), urothelial carcinoma (UC) and renal cell carcinoma (RCC), underlining the prognostic role and therapeutic impact of molecular pathways, discussing recent clinical data published in the last three years, and investigations currently in progress, with a focus on the strengths and weaknesses of clinical applicability of this approach.

To shed light on these issues, we conducted an electronic PubMed search focusing on “circulating tumor cells” combined with keywords such as “liquid biopsy”, “molecular pathways”, “genitourinary cancers”, “prostate cancer”, “bladder cancers”, “renal cell carcinoma”, “targeted therapies”, and “immune checkpoint inhibitors”.

## 2. Prostate Cancer

### 2.1. Genomic Landscape and Potential Targets

Several genetic alterations have been identified in different stages of PCa. Generally, in the early phase, PCa growth depends mostly on androgen stimulation and is highly responsive to androgen deprivation therapy (ADT) (hormone sensitive phase). In its natural history, PCa develops resistance to hormone inhibition (castration resistant prostate cancer (CRPC)). Therapies adopted in this phase are new androgen receptor signaling (ARS) inhibitors such as abiraterone acetate or enzalutamide, or other chemotherapeutic agents. CRPC can transdifferentiate and express neuroendocrine (NE) markers, such as chromogranin and synaptophysin. In this case, co-expression of these markers on CTCs can raise the uncertainty of NE differentiation that has direct clinical consequence considering the potential benefit of platinum-based chemotherapy [8].

Moreover, *Aurora kinase A* (AURKA) amplification and overexpression have been found in a series of small cell PCa. Patients with AURKA alterations can be potential candidates for targeted therapy with Aurora kinase inhibitor, Alisertib [9].

Molecular pathways currently under investigation for their potential predictive value and implications as therapeutic targets are DNA repair genes such as poly-ADP ribose polymerase (*PARP*)*1* and *PARP2*, homologous recombination (HR) system genes (in particular *BRCA1*, *BRCA2*, and *ATM*), and mismatch repair genes [10,11]. A phase II study is currently evaluating the efficacy of pamiparib in metastatic CRPC (mCRPC) in patients with CTCs with homologous recombination deficiency (CTC-HRD) (NCT03712930).

**Figure 1:**
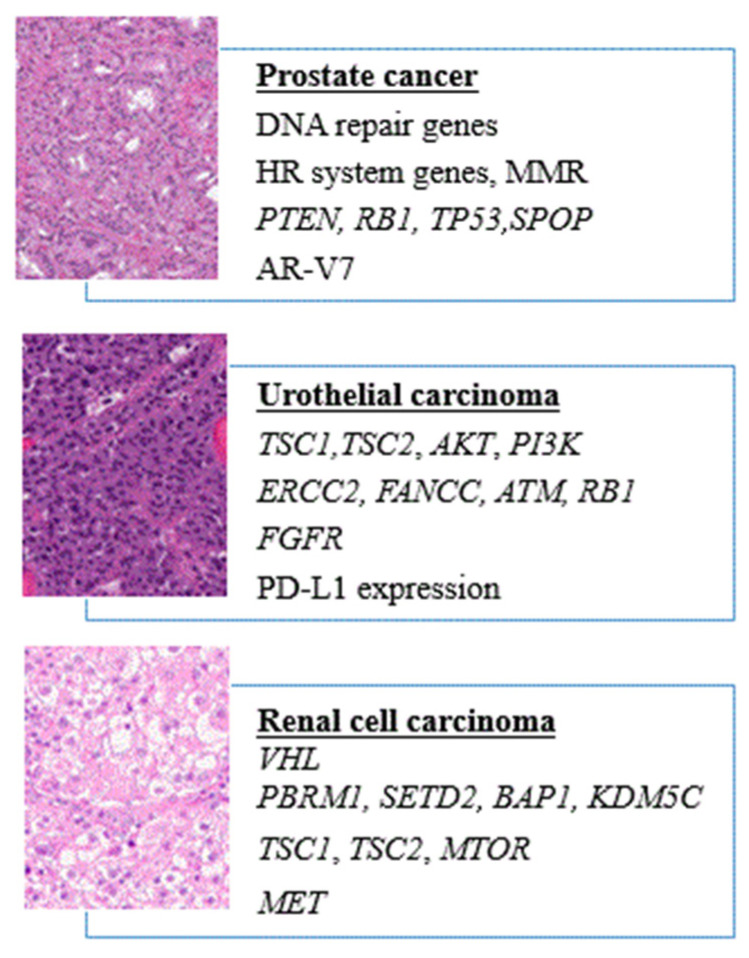
Potential prognostic and predictive genes and surface markers detectable in circulating tumor cells (CTCs) in prostate carcinoma, urothelial carcinoma, and renal cell carcinoma.

DNA repair mutations are present in 15–30% of mCRPC and are associated with poor prognosis and aggressive behavior. Prostate cancer patients with defects in DNA repair genes respond to poly-ADP ribose polymerase (PARP) inhibitors and are sensitive to platinum chemotherapy [12,13,14,15]. On the other hand, alterations in mismatch repair genes are associated with response to immunotherapy, like anti-programmed death 1 (PD1) or anti-PD-L1 drugs [16]. More commonly mutated genes such as *PTEN*, *RB1*, and *TP53*, are associated with poor prognosis, increased risk of recurrence and death, and are frequently altered in CRPC compared to primary PCa. The presence of mutations in these genes have also been associated with poor response to hormonal treatments and ARS inhibitors [17,18,19,20,21]. On the contrary, *SPOP* mutations are associated with more favorable prognosis and higher response rate to abiraterone, although its alterations are present only in 5% of mCRPC [22,23]. In the end, androgen receptor (AR) splice variants, in particular splice variant 7 (AR-V7), have been correlated with abiraterone and enzalutamide resistance by the induction of independent constitutive receptor activation [24,25,26] (Figure 1). Therefore, finding these genetic alterations in CTCs can help clinicians choose the right, tumor-tailored therapy and detect early resistance to treatment. In this setting, liquid biopsy offers some advantages in the detection of prognostic and predictive biomarkers, with the possibility of molecularly characterizing the tumor in its different phases to avoid repeated tissue biopsies on our patients.

### 2.2. Selection of Published and Ongoing Clinical Trials

Here we present a selection of published clinical studies investigating the role of CTCs in PCa, followed by some ongoing trials. The landmark trial by de Bono and colleagues was prospectively conducted in 231 patients affected by mCRPC starting a new line of chemotherapy [27]. The authors demonstrated that a CTC count >5 per 7.5 mL of blood at any time during the course of disease was associated with poor outcome, was predictive of a shorter progression-free survival (PFS), and resulted in the strongest independent predictor of overall survival (OS), when matched to prostate specific antigen (PSA) changes after treatment [27]. A recent large analysis used individual patient data from five prospective phase III randomized trials. A total of 6081 mCRPC patients treated with different hormone therapies in four trials (COU-AA-301 [28], AFFIRM [29], ELM-PC-4 [30], ELM-PC-5 [31]), with only one trial concerning taxane-based chemotherapy (COMET-1 [32]), was analyzed using the CellSearch assay. The results underlined that patients with CTC count >1 at baseline and 0 at week 13 (CTC0 end point) had improved survival [33]. Concerning the hormonal-resistance biomarker AR-V7, the multicenter prospective PROPHECY study dealing with mCRPC patients under abiraterone or enzalutamide recently confirmed and validated the value of AR-V7 in CTCs. From this study, AR-V7 positive CTCs were independently associated with worse survival outcome, both PFS and OS [34]. A phase II multicenter study evaluating response to Cabazitaxel in mCRPC patients with AR-V7 positive CTCs is ongoing (NCT03050866). Another ongoing phase II clinical trial seeks to define the association of AR-V7 status in CTCs and AR gene alterations with PSA response to docetaxel and enzalutamide (NCT03700099). AR-V7 expression has also been evaluated on tumor clusters by Okegawa et al. The multivariable analysis concluded that presence of pre-therapy CTC cluster and presence of CTC cluster AR-V7 negative were independently associated with a poor PFS and OS in abiraterone- and enzalutamide-treated patients [35].

Recently, some researchers have shed light on the clinical role of prostate-specific membrane antigen (PSMA) expression in CTCs from a small cohort of mCRPC patients under treatment. They proved that PSMA was correlated with poorer treatment response, shorter OS, and was inversely associated with PSA changes, thus postulating PSMA-positive CTCs as an independent poor prognostic biomarker in such patients [36]. These findings are useful in order to submit a patient to a PSMA PET-CT and also as a predictive biomarker for PSMA-targeted radionuclide therapy with 177Lu-Labeled PSMA-617 and as an immunotherapeutic target [37,38].

However, mRNA extraction followed by reverse transcription polymerase chain reaction (RT-PCR) for detecting the expression of PSMA does not discriminate between the different pattern of expression of this transmembrane protein that can be seen on the surface of the cell, at the cell membrane level, and/or in the cytoplasm with immunohistochemical techniques. Both the imaging and therapy applications of PSMA are mostly due to the extracellular expression of the protein [39,40].

As regards investigations in progress, a phase I dose de-escalation trial in patients with metastatic PC and unfavorable CTC count (>5/7.5 mL of whole blood) is evaluating the monoclonal antibody (mAb) called J591 against the extracellular domain of PSMA, in an attempt to define the effect of mAb Hu-J591 on CTCs. Importantly, the primary outcome measure of this trial deals with the tumor response at every dose level as defined by conversion from an unfavorable CTC count at baseline to a favorable CTC count (<5/7.5 mL) (NCT02552394). In addition, a prospective cohort study in patients with metastatic PC is aimed at exploring changes in expression of four immune checkpoint biomarkers (PD-L1, PD-L2, B7-H3, and CTLA-4) on CTCs via the CellSearch method. The study enrolled patients planning to start immunotherapy with new hormonal agents (NHAs) (group A), or without NHAs (group B), or ADT (group C, in metastatic castration-sensitive PC), or progressing to NHA and candidates for radium-223/chemotherapy (group D) (NCT02456571) (Table 1 and Table 2).

In addition to enumerating CTCs, Faugeroux et al. performed whole exome sequencing on CTCs in mPC. They found that epithelial CTCs share mutations with matched metastasis biopsies, while CTC-exclusive mutations were identified in genes involved in invasion, DNA repair, cytoskeleton, and tumor-driver genes and were found in both epithelial and nonepithelial CTCs [49].

## 3. Bladder Cancer

### 3.1. Genomic Landscape and Potential Targets

The recent advances in genome sequencing, as well as transcriptome analysis, have considerably changed the molecular classification of bladder cancer (BC) providing new insights in potential target genes and pathways. The recently published consensus on molecular classification of muscle-invasive bladder cancer (MIBC) identified six molecular classes. About 35% of MIBC is classified as basal/squamous, 24% as luminal papillary, 8% as luminal non-specified, 15% as luminal unstable, 15% as stroma-rich, and 3% as neuroendocrine-like. This molecular classification is useful to stratify patients for prognosis, prediction of response, and as a potential tool for personalizing neoadjuvant therapy selection [50,51,52,53].

This classification is based on shared RNA expression patterns or specific genomic alterations using large-scale expression and sequencing data sets that are not applicable to single cell detection and consequently, liquid biopsy. According to The Cancer Genome Atlas (TCGA), potentially actionable mutations are present in nearly 68% of BC. These can be found in the primary tumor and in CTCs, and this is of great importance since studies showed that within a patient most of the genetic alterations are not shared across multiple tumor sites [54].

Recurrent alterations have been found in the phosphoinositide 3-kinase (PI3K)-AKT-mammalian target of rapamycin (mTOR) and receptor tyrosine kinase (RTK)-MAPK pathways. Potentially targetable alterations in these pathways include those in tuberous sclerosis complex (*TSC*) 1 or *TSC2* (9%), *AKT* (10%), and phosphoinositide 3-kinase (*PI3K*) (17%) [44]. *TSC1* mutations seem to confer mTOR inhibitor sensitivity [55]. Mutations in the DNA repair pathway genes *ERCC2*, *FANCC*, *ATM*, and *RB1* are associated with complete pathological response after neoadjuvant chemotherapy and need to be further considered for their utility in the therapeutic strategy [41,42,56,57]. Moreover, great enthusiasm in the clinical community has been elicited by another targetable gene fibroblast growth factor receptor (*FGFR*)3 [43].

The majority of *FGFR3* alterations in BC have been found in luminal papillary tumor subtype and in non-muscle invasive bladder cancer (NMIBC) and are often associated with better outcome [45]. Mutations in *FGFR* gene account for approximately 20% of patients with recurrent or refractory BC [58]. Accelerated approval by the FDA was recently granted to erdafitinib for patients with advanced UC with alterations of *FGFR2* or *FGFR3* who have progressed on platinum-based chemotherapy given the 40% confirmed response to erdafitinib [59]. To date, no published or ongoing studies are present on FGFR expression in CTCs, but the detection of CTCs with FGFR2 expression by FACScan has been applied in patients with gastric cancer, thus demonstrating the technique’s feasibility [60].

Of great interest is also the possibility to investigate PD-L1 expression status on CTCs. Presence of high PD-L1^+^/CD45^−^ CTCs and low burden of apoptotic CTCs in MIBC patients have been associated with worse OS. Although the feasibility to detect and identify PD-L1 positive CTCs has been demonstrated, more studies are needed to assess the predictive value of this method in response to immunotherapy [61,62,63].

### 3.2. Selection of Published Clinical Trials

Urothelial carcinoma (UC) is a cancer in which no biomarker has still been validated for monitoring the disease course, both for early phase and advanced stage. Some evidence has documented high expression of CTCs in metastatic UC [64,65]. Furthermore, in patients with nonmetastatic UC, CTCs are detectable in almost 25% of cases [66]. In this regard, using patients with clinically localized BC, two large prospective trials have studied the significance of CTCs with the CellSearch system and generated concordant results. The first trial showed that in patients considered candidates for radical cystectomy, preoperative CTCs were significantly correlated with higher risk of recurrence as well as cancer-specific and overall mortality [66]. The second trial, in which high-risk T1G3 BC patients underwent conservative surgery, highlighted that the detection of CTCs significantly predicted both decreased times to first local recurrence and shorter PFS. The authors concluded that CTCs can select patients in early stages as having systemic disease ab initio and accordingly are likely suitable for systemic therapy [67]. In 2019, the same group published results of a single-center prospective study designed to explore the impact of CTCs on cancer-specific survival (CSS) and OS in 102 high grade (HG) T1 bladder cancer patients. They demonstrated that even a single CTC is predictive of shorter CSS and OS [68].

Conversely, other investigators did not find any detection of CTCs in localized BC [69,70], leading to controversy surrounding the role of CTCs in nonmetastatic BC patients. A recent meta-analysis assessed a total of 2161 BC patients at different disease stages, correlating the presence of CTCs with tumor stage, histological grade, regional lymph node metastasis, and metastases, indicating that CTCs are more easily detected in more advanced stages of BC. Furthermore, patients CTC-positive versus CTC-negative exhibited poorer cancer specific survival, PFS, disease-free survival, and OS [71]. Along this line, CTC assessment using the CellSearch System in 33 patients with metastatic UC underlined a higher number of CTCs in patients with more than two metastatic sites compared to those with <1 metastatic site [72]. Of interest, in a pilot study with AdnaTest and multiplex-PCR as new methods for interrogating blood samples by 31 metastatic UC patients under front-line chemotherapy, the authors observed that CTC changes occurring during chemotherapy were associated to better survival prediction in terms of PFS and OS than CTC measurement at fixed time points [72]. CTCs were also detected in about 25% of patients with variant UC histology before radical cystectomy. Even in the variant histology group, patients with CTCs experienced a worse outcome compared to patients without CTCs [73].

More recently, Bergmann et al. investigated PD-L1 expression on CTCs in the blood of patients with advanced UC through the CellSearch System. PD-L1 expression in ≥1 CTC was found in 63% of CTC-positive samples. CTC detection and presence of CTCs with moderate or strong PD-L1 expression was associated with poor survival [74]. PD-L1 expression on CTCs was also demonstrated by Anantharaman et al. in both Cytokeratin (CK)+ and CK− CTCs in patients with metastatic bladder cancer. PD-L1 expression on CTCs might facilitate immune escape in the blood stream conferring a survival advantage and promoting metastatic spread [75]. Taking into account all available evidences, it is worthy of mention that there is a paucity of CTC data in terms of large patient population in metastatic UC, contrary to the neo-adjuvant UC setting. Pure investigations currently ongoing in this disease are scarce.

## 4. Renal Cell Carcinoma

### 4.1. Genomic Landscape and Potential Targets

The pathological classification of RCC has three common subtypes: clear cell RCC (ccRCC) is the most frequent, followed by papillary RCC, and chromophobe RCC. von Hippel-Lindau (*VHL*) tumor suppressor gene is the most commonly alterated gene in ccRCC [76], mesenchymal epithelial transition receptor (*MET*) gene alteration is frequently found in sporadic papillary type 1 RCC, while sporadic papillary type 2 RCC is characterized by cyclin-dependent kinase inhibitor 2A (*CDKN2A*), SET domain containing 2 (*SETD2*), neurofibromin 2 (*NF2*), Cullin-3 (*CUL3*), telomerase reverse transcriptase (*TERT*) mutations, and chromosomes alterations [77]. Chromophobe RCC usually harbors combined losses involving most or all of chromosomes 1, 2, 6, 10, 13, 17, and 21 and mutations in *TP53* (32%) and *PTEN* (6%) [78]. Other RCC histotypes are characterized by definite genetic alterations. Microphthalmia-associated transcription (MiT) family translocation RCC is defined by transcription factor binding to IGHM enhancer 3 (*TFE3*) (Xp11.2) and transcription factor EB (*TFEB*) (t(6;11)) translocation and succinate dehydrogenase-deficient RCC [46,47]. In the rare RCC category, collecting duct carcinoma (CDC) harbors mutations in *NF2*, *SETD2*, *SMARCB1*, *FH*, and *CDKN2A* genes [79] while renal medullary carcinoma is distinctively characterized by loss of SMARCB1/INI1 tumor suppressor protein [48]. Searching these specific mutations in liquid biopsies could help clinicians in monitoring the patients during follow-up and detect residual disease after nephrectomy.

Comprehensive molecular characterization of RCC identified numerous mutations associated with prognosis and response to therapy [80,81,82,83,84,85,86,87]. Polybromo-1 (*PBRM1*), *SETD2*, BRCA1-associated protein-1 (*BAP1*), and lysine demethylase 5C (*KDM5C*) alterations are associated with poor prognosis [81]. Mutations in *TSC1*, *TSC2*, and mammalian target of rapamycin (*mTOR*) correlate with sensitivity to everolimus [82,83,84]. Differences in PFS have been demonstrated in *PBRM1*-mutated patients treated with sunitinib or atezolizumab plus bevacizumab compared to atezolizumab alone [86]. In patients with confirmed *MET*-driven papillary RCC the *MET*-inhibitor savolitinib has shown promising activity [88,89].

Spatial and temporal heterogeneity are distinctive properties of RCC and the potential cause of the development of acquired or primary resistance. Primary resistance to angiogenesis inhibition has been ascribed to *HIF-2α* expression in VHL deficient tumors and to inhibition of apoptosis by synthesis of B-cell lymphoma-2/XL (*Bcl-2*/*XL*) [90,91]. To overcome VEGF/VEGFR blockade, cancer cells acquire different pathways to increase angiogenesis such as PDGF/PDGFR and MET pathways [92]. Overexpression of *FGFR* has also been linked to the development of sunitinib resistance [93]. Knowing this, the clinician can decide to change the therapeutic strategy or to combine multiple target drugs in order to overcome potential resistance.

### 4.2. Selection of Published Clinical Trials

Literature data indicate that the detection of CTCs in patients affected by RCC is an event occurring early during the disease course and is likely associated with more aggressive tumor features [94]. Some researchers interrogated 214 RCC patients and collected peripheral blood samples perioperatively and during adjuvant immunotherapy. A semi-automated immunomagnetic depletion assay using the magnetic-activated cell sorting (MACS) method was used. The findings importantly underlined that CTCs were detected in 37% of patients, and 62% developed distant metastases or died because of RCC within two years [94]. In addition, a perioperative prospective detection of CTCs in 60 RCC patients treated with different surgical modalities was recently published. The authors found a significantly greater number of CTCs after open radical nephrectomy (RN) than after laparoscopic procedures, confirmed performing a multivariate analysis, thus speculating the need for more stringent clinical monitoring after RN [95]. A molecular characterization of CTCs collected from 10 metastatic RCC patients as a post hoc analysis from the TARIBO trial [96] was recently published. Two patients with detectable epithelial CTCs prior to systemic treatment start exhibited short PFS, however the positivity rate of non-epithelial CTCs was higher than conventional/epithelial CTCs. Again, CTC analysis at single-cell level in a case study showed genomic alterations (9p21.3 loss) known as drivers of metastases, thus potentially triggering cancer progression [97].

Metastatic patients with RCC and UC starting ICIs are being prospectively examined in a cohort study and divided into group A and group B, respectively. CTC detection as primary outcome measure is planned in blood samples at baseline, 4 weeks, and upon disease progression, while CTC changes over time and correlation between CTCs and tumor response are assessed as secondary outcomes. This study aims to profile CTCs under ICIs through characterization of targets such as PD-1, PD-L1, CTLA-4, CD27, OX40, or LAG3 (NCT02978118).

## 5. Strengths and Weaknesses of CTCs

Compared to tissue biopsies, CTCs better reflect tumor heterogeneity because they originate from different tumor sites, giving an overview in the collection of genetic tumor alterations and in the presence of different subclones. Moreover, they offer the possibility to investigate how tumor cells become resistant to therapy since they can be evaluated longitudinally during the course of therapy, in a non-invasive way [98,99]. Contrary to circulating DNA, CTCs offer the possibility to perform certain assays like whole exome sequencing, splice variants analysis, information at single-cell level, and functional assays. CTCs can also be cultured to evaluate drug resistance in vitro or in vivo [100]. However, isolation of CTCs is technically difficult due to the extremely small number of CTCs in patient blood (one CTC per billion blood cells) and short half-life. Current isolation methods rely on physical properties such as dimensions, elasticity, density, and expression of epithelial markers (epithelial cell adhesion molecule (EpCAM)). The sensitivity of this system is reduced by the absence or the loss of cytokeratin expression on tumor cells, thus becoming undetectable during CTC isolation. Sized-based methods of isolation of CTCs in whole blood are often impaired by clotting of filter pores by blood cells. A new combination of epithelial markers and the adoption of new methods of detection based on multi-parameter immunofluorescence microscopy (MPIM) have improved the sensitivity and overcome this issue [101,102,103,104]. Capture of CTM is even more difficult due to the absence of specific biomarkers on their surface, with them being covered by macrophages, platelets, and stromal cells. Microfluidic devices based mainly on size differences have been developed to overcome this obstacle. [105,106,107,108,109,110,111]. Polymerase chain reaction (PCR)-based assays have become the most widely used alternative to immunology-based techniques. This technique allows to detect specific mRNAs expressed by viable CTCs [111]. (Table 3 and Table 4)

## 6. Potential Application

The first obstacle for the clinical application of precision oncology is to identify and select molecular biomarkers able to predict outcome, sensitivity or resistance to a specific drug or combination therapies, or specific drug-related adverse reactions [115,116].

In the early phase, presence of CTCs identifies more aggressive tumors that could benefit from a close follow-up program and perhaps a more aggressive treatment at the time of clinical recurrence. Specifically, in the case of PCa, early identification of castration-resistant status during anti-androgen therapy could help clinicians to avoid inappropriate treatment. Additionally, the acquisition of new somatic mutation during treatment such as DNA repair genes alterations or other targetable genes might drive the selection of a more customized treatment plan. In UC scenario, CTC detection after surgery could predict those patients that could benefit more from perioperative chemotherapy since they are at a greater risk of disease recurrence. Even in NMIBC, CTCs may be of help clinicians in identifying those patients with shorter time to recurrence or progression, or potential candidates for early systemic therapy. Moreover, in a pre-surgical setting, the presence of CTCs in patients initially classified into locally confined (stage ≤II) disease is associated with an increase in stage (stage III, IV) after surgery [117]. Hence, the assessment of CTC status could be helpful in selecting patients who could benefit from neoadjuvant chemotherapy. In RCC, CTCs can be assessed to detect residual or micrometastatic disease after surgery, to monitor tumor response during therapy, to understand mechanisms of resistance, and to identify new targetable mutations that can emerge during treatment selection. It also important to consider that the majority of information on predictive markers such as PD-L1 or genetic characterization are obtained from the primary tumor, usually a section of the tumor mass. Patients generally undergo multiple lines of therapy during which the tumor undergoes genetic alteration and clone selection. The application of CTCs may overcome the tumor heterogeneity and time evolution issues offering an overview of the tumor biology [118]. All these potential applications take into account not just the quantitative assessment of CTCs, but more importantly their genetic content and surface biomarkers. Moreover, CTCs can be targets for anticancer therapy. New therapeutic strategies should be directed towards preventing cancer dissemination through the elimination of CTCs in vivo. Kim et al. tested this hypothesis in mice models using photodynamic therapy to specifically eliminate green fluorescent protein (GFP)-expressing CTCs [119]. The elimination of CTCs demonstrated to be effective in suppressing distant metastasis and increasing the survival of the tumor-bearing mice. In the end, CTCs offer the possibility to develop CTC-derived 3D organoid models that are of outstanding importance to identify driver genes through manipulation with inhibitors, retrovirus, and CRISPR/Cas9 approaches [120] and to discover the molecular basis of drug response [121].

In conclusion, CTC detection and characterization have shown potential to guide cancer treatment and provide valuable information for patient-tailored therapies; however, the molecular and immunohistochemical analysis of CTCs requires further studies and explorations along with the development of new advanced techniques that can be applicable in practice and cost-effective.

## Figures and Tables

**Table 1:** Selected studies on CTCs in prostate cancer, urothelial carcinoma, and renal cell carcinoma.

Reference	Study Design	Patients and Therapy	Results
de Bono et al. [20]	Multicenter prospective study	231 mCRPC patients starting a new line of chemotherapy	Better OS in favorable group (<5 CTCs per 7.5 mL). Post-treatment decrease in CTC number correlated with a better OS compared to patients whose CTC number remained ≥ 5.
Heller et al. [26]	Analysis of 5 prospective randomized phase III trials	6081 patients with mCRPC	CTC count before treatment start and CTC conversion from above to below 5 CTCs is a biomarker to differentiate OS for 13-week responders and non-responders.
Armstrong et al. [27]	Multicenter prospective validation study	118 high-risk mCRPC patients treated with abiraterone or enzalutamide	CTC nuclear-specific AR-V7 protein assay is independently associated with worse PFS and OS.
Nagaya et al. [28]	Observational study	56 CRPC patients who progressed on therapy and switched to new treatment	Shorter median PSA, PFS, and OS in the PSMA-positive CTC cohort. PSMA expression was associated with poorer response, and shorter PSA, PFS, and OS.
Rink et al. [41]	Prospective study	100 consecutive UC patients treated with radical cystectomy	Higher risk of disease recurrence and cancer-specific and overall mortality in CTC-positive patients.
Gazzaniga et al. [42]	Prospective single center trial	102 high-risk T1G3 bladder cancer	CTCs were detected in 20% of patients and predicted shorter time to first recurrence and time to progression.
Zhang et al. [43]	Meta-analysis of 30 studies	2161 urothelial cancer patients	CTC-positive was significantly associated with tumor stage, histological grade, metastasis, regional lymph node metastasis, and poor OS, PFS/DFS, and CSS.
Gallagher et al. [44]	Observational study	33 patients with metastatic UC	Higher number of CTCs was seen in patients with two or more sites of metastases.
Fina et al. [45]	Single-center, prospective study	31 patients mUC receiving first-line MVAC chemotherapy were collected at baseline (T0) and after 2 cycles (T2)	Changes in CTC better predicted 3-year PFS and OS compared to CTC status evaluated at single time points. No association was found between CTCs and objective response to MVAC.
Bluemke et al. [46]	Observational study	154 RCC	Presence of CTCs correlates with lymph node metastasis, presence of synchronous metastases, and poor OS.
Haga et al. [47]	Single center study	60 RCC patients underwent LRN, LPN, ORN, and OPN	ORN resulted in significantly perioperative changes in CTCs and in a greater number of postoperative CTCs compared to LRN, LPN, and OPN.
Cappelletti et al. [48]	Observational study	21 blood samples serially collected from 10 patients with metastatic RCC entering the TARIBO trial	Two CTC subpopulations were identified: epithelial CTCs (eCTCs) and non-conventional CTCs (ncCTCs) lacking epithelial and leukocyte markers. With a threshold ≥1 CTC/10 mL of blood, eCTCs were found in 28% of samples, ncCTCs in 62%, and both CTC types in 71%.

CTCs: circulating tumor cells; mCRPC: metastatic castration resistant prostate cancer; OS: overall survival; PFS: progression-free survival; PSMA: prostate specific membrane antigen; PSA: prostate specific antigen; UC: urothelial carcinoma; DFS: disease-free survival; CSS: cancer specific-survival; RCC: renal cell carcinoma; LRN: laparoscopic radical nephrectomy; LPN: laparoscopic partial nephrectomy; ORN: open radical nephrectomy; OPN: open partial nephrectomy; MVAC: methotrexate, vinblastine, doxorubicin, and cisplatin.

**Table 2:** Ongoing trials on CTCs in genitourinary tumors.

Trial ID	Primary Outcome	Disease	Treatment	Method
NCT02978118	Number of patients with detectable CTCs	UC and RCC	Immune checkpoint inhibitors	Not specified
NCT02552394	Determine the effect of mAb Hu-J591 on reducing CTCs	Advanced prostate cancer (PCa)	J591	CellSearch
NCT02456571	Expression of immune checkpoint biomarkers (PD-L1, PD-L2, B7-H3, and CTLA-4) on CTCs	Metastatic PCa	Sipuleucel-T or abiraterone acetate or enzalutamide or androgen deprivation therapy (ADT)	CellSearch
NCT03712930	Efficacy of pamiparib in patients with CTCs with homologous recombination deficiency (CTC-HRD)	mCRPC	Pamiparib	Not specified
NCT03700099	Correlate AR-V7 status in CTCs and PSA response decline	mCRPC	Sequential treatment with docetaxel and enzalutamide	Not specified
NCT03050866	Correlate AR-V7 CTCs with response to cabazitaxel	mCRPC	Cabazitaxel	Not specified

**Table 3:** Currently available CTC methods of enrichment and detection.

Technology	Advantages	Disadvantages	Potential Solutions
Size-based microfluidic isolation	Easy and rapid; feasible for epithelial cell adhesion molecule (EpCAM)-negative CTCs and for a wide range of tumors	Loss of smaller CTCs or clotting of filter pores by blood cells	Fluid-assisted separation technology, combined methods (CTC-iChip) [106,107,108]
Density gradient centrifugation	Operability; feasible for EpCAM-negative CTCs; Elimination of lymphocytes and mononuclear cells	Loss of some CTCs, lack of specificity	Combination with other methods (i.e., automated immunofluorescence staining) [109]
Immunoaffinity	High purity, visual confirmation of CTCs	Costly, absence of standardized markers	Use of multiple antibodies simultaneously [110]
Microfluidics sorting device	High recovery and efficiency; potential to recover CTCs for molecular or IHC characterization	Absence of standardized methods; high technical requirement	Combination with other methods (i.e., RT-PCR based selection) [111]

**Table 4:** Antibodies used for CTC detection in genitourinary tumors.

Antibodies for CTC Detection	Application	Findings
EpCAM and CD45 (CellSearch^®^ System)	Epithelial tumors	EpCAM negative tumor cells may not be detected—lack of specificity for tumor cells. Nonmalignant epithelial cells are false positive
Citokeratins (CK8/18CK-19/CK-20)	Epithelial tumors	Cytokeratin (CK) negative tumor cells may not be detected—poor specificity for tumor cells
PSMA/HER2 (+size selection)	Prostate cancer	High cell capture efficiencies and highly pure captured cell [110]
EpCAM, HER-2 and PSA	Prostate cancer	High cell capture efficiency (tested on cell lines) [112] and
PSMA/CD45	Prostate cancer	higher sensitivity compared to CellSearch [113]
CA9 and/or CD147	Clear cell renal cell carcinoma (ccRCC)	CA9 and/or CD147 expression in 97.1% of patients with ccRCC tumors (EpCAM detected only 18.6%), poor specificity [101]
CA9 (mAbG250)	Clear cell renal cell carcinoma	Lack of specificity, CAIX can also be expressed in hypoxic or necrotic tissues regardless of their tumor origin [114]
